# Implicit Motivational Processes Underlying Smoking in American and Dutch Adolescents

**DOI:** 10.3389/fpsyt.2014.00051

**Published:** 2014-05-19

**Authors:** Helle Larsen, Grace Kong, Daniela Becker, Janna Cousijn, Wouter Boendermaker, Dana Cavallo, Suchitra Krishnan-Sarin, Reinout Wiers

**Affiliations:** ^1^Addiction Development and Psychopathology (ADAPT)-Laboratory, Developmental Psychology Research Program, University of Amsterdam, Amsterdam, Netherlands; ^2^Research Priority Area Yield, University of Amsterdam, Netherlands; ^3^Department of Psychiatry, Yale School of Medicine, New Haven, CT, USA; ^4^Social Psychology Research Program, University of Amsterdam, Amsterdam, Netherlands

**Keywords:** smoking, adolescents, cognitive biases, risk taking, inhibition, working memory

## Abstract

**Introduction:** Research demonstrates that cognitive biases toward drug-related stimuli are correlated with substance use. This study aimed to investigate differences in cognitive biases (i.e., approach bias, attentional bias, and memory associations) between smoking and non-smoking adolescents in the US and the Netherlands. Within the group of smokers, we examined the relative predictive value of the cognitive biases and impulsivity related constructs (including inhibition skills, working memory, and risk taking) on daily smoking and nicotine dependence.

**Method:** A total of 125 American and Dutch adolescent smokers (*n* = 67) and non-smokers (*n* = 58) between 13 and 18 years old participated. Participants completed the smoking approach–avoidance task, the classical and emotional Stroop task, brief implicit associations task, balloon analog risk task, the self-ordering pointing task, and a questionnaire assessing level of nicotine dependence and smoking behavior.

**Results:** The analytical sample consisted of 56 Dutch adolescents (27 smokers and 29 non-smokers) and 37 American adolescents (19 smokers and 18 non-smokers). No differences in cognitive biases between smokers and non-smokers were found. Generally, Dutch adolescents demonstrated an avoidance bias toward both smoking and neutral stimuli whereas the American adolescents did not demonstrate a bias. Within the group of smokers, regression analyses showed that stronger attentional bias and weaker inhibition skills predicted greater nicotine dependence while weak working memory predicted more daily cigarette use.

**Conclusion:** Attentional bias, inhibition skills, and working memory might be important factors explaining smoking in adolescence. Cultural differences in approach–avoidance bias should be considered in future research.

## Introduction

Adolescent tobacco use is not only related to negative health outcomes and smoking later in life, but also to increased risk for developing other addictive behaviors (1, 2). In many countries, including the Netherlands and the United States, in which this study took place, onset of smoking and the transition into regular smoking are often observed in adolescence. In the Netherlands, 24% of the adolescents between 15 and 19 years old were daily smokers (3). In the United States, 18% of the adolescents between 13 and 18 years old reported cigarette smoking in the past month (4).

As relatively little is known about the motivational mechanisms underlying adolescent smoking, dual-process models of addiction may provide a better understanding of adolescent smoking behavior. This, in turn, could refine early prevention and intervention strategies. Dual-process theories posit that behavior is the outcome of an imbalance between two qualitatively different types of processes; on the one hand reflective processes and on the other hand impulsive processes (5, 6). Reflective processes are typically slow, explicit, and have a limited capacity, whereas impulsive processes are typically triggered automatically and concern emotional and motivational processes (7, 8). From this perspective, addiction may then be the outcome of strong drug-oriented impulsive processes combined with relatively weaker ability to control these processes. Motivational processes form part of the impulsive processes, which become sensitized after repeated drug use (5, 7, 9). According to the incentive–sensitization theory of Berridge and Robinson (10), being exposed to a drug-related cue activates the mesolimbic dopamine system and increases dopamine levels in the brain (i.e., resulting in increased “wanting,” which may at some point dissociate from “liking”). The central tenet of this theory is that with use becoming more regular, the brain learns that substance-related cues are associated with the rewarding effects. When subsequently exposed to a cue, incentive salience is attributed to the cue through activation of the mesolimbic dopamine system, ultimately leading to a motivation (which may be subjectively experienced as craving) to consume the drug (11).

In human studies, incentive–sensitization has been related to cognitive biases, such as attentional bias (9), approach bias (10, 12), and implicit or automatically activated memory associations (13, 14). Note that these associations have been primarily theory-based, and cognitive biases could also be related to habit-formation [see Stacy and Wiers (15) for an overview]. Cognitive biases related to smoking have mainly been examined in student populations and adults and only a few studies have examined cognitive biases in adolescents. Attentional bias is the tendency to allocate attention to drug-related cues in the environment. For example, smoking urges may be triggered by the sight of cigarettes or others’ smoking. Several studies demonstrate attentional bias toward smoking-related stimuli among young adult and adult smokers (16–23). Approach bias is the relatively automatically triggered tendency to approach rather than avoid drug-related stimuli. Studies have found approach biases toward marijuana in young adults (24, 25), and toward alcohol use in at-risk adolescent drinkers (26, 27), and young adults (12, 28). However, as yet studies on approach bias and smoking have only been conducted in young adults and adults. These studies found evidence for approach bias among young adult and adult smokers (20, 22, 29–31). Implicit associations reflect associations between mental representations in memory. Previous studies demonstrate positive implicit associations with smoking in young adults (32, 33), but also negative associations with smoking in adults (34) and young adults (35). A recent study also found negative implicit associations with smoking in both adult smokers and non-smokers (36).

To further understand adolescent smoking, it is important to investigate whether and how these cognitive biases are related to adolescent smoking and non-smoking behavior. To date, few studies have investigated cognitive biases in adolescents and most have focused on alcohol consumption (37–40). This study aimed to investigate attentional bias, approach bias, and implicit associations in adolescent smokers and non-smokers.

Other factors related to executive functioning and personality characteristics may also influence smoking behavior in adolescents. For instance, smoking behavior in adolescents and young adults has been linked to weaker working memory [e.g., Ref. (41, 42)], more risk taking (43, 44), and weak inhibition skills [e.g., Ref. (5)]. Hence, the current study additionally investigated the relative predictive value of cognitive biases (i.e., approach bias, attentional bias, and implicit associations), working memory, risk taking, and inhibition skills on adolescent smoking behavior.

Despite the relatively large number of studies on cognitive biases and substance use, there is a paucity of studies using the same assessment method investigating cognitive biases and substance use in adolescence across different cultural contexts. This is important since cognitive biases have been shown to be sensitive to context effects [e.g., Ref. (45, 46)]. We, hence, took a cross-national approach and investigated attentional bias, approach bias, and implicit associations in American and Dutch adolescent smokers and non-smokers. Given that according to the dual-process model, the implicit processes involved in addiction should be similar in American and Dutch adolescents, we hypothesized smoking biases to be comparable between Dutch and American smokers and non-smokers. Based on previous research, we generally expected smokers to demonstrate stronger biases and positive associations toward smoking than non-smokers. Within the group of smokers, we expected positive associations between cognitive biases, risk taking, and smoking outcomes (that is amount of daily cigarette use, nicotine dependence) and negative associations between working memory, inhibition skills, and smoking outcomes.

## Materials and Methods

### Participants

Participants were 79 Dutch adolescents [smokers (*n* = 41) and non-smokers (*n* = 38)] and 46 American adolescents [smokers (*n* = 26) and non-smokers (*n* = 20)] between the age of 13–18 years [*M* = 16.08, standard deviations (SD) = 1.32]. One participant reported to be 20 years old and was not included in further analyses. Inclusion criteria for adolescent smokers were that they had smoked more than five cigarettes a day during the preceding 6 months. Non-smokers were those who reported never having smoked a cigarette in their lifetime, with biochemical verification of non-smoking status (US: urine cotinine levels of <50 ng/ml at the time of assessment, NL: CO breath <0.40). Exclusion criteria were depressive tendencies assessed with Beck depression inventory (BDI-II, 47) and the use of psychoactive medicine <2 months. No participants were excluded from participation based on these criteria. The ages of cigarette initiation [*t*(63) = −0.17, *p* = 0.87] and daily smoking [*t*(63) = −0.36, *p* = 0.72] did not differ between Dutch and American adolescents. On average, they were 13 years old (SD = 1.50) when they had their first cigarette and 14 years old (SD = 1.41) when they became daily smokers.

### Procedure

In both studies, once we obtained permission from the school boards, adolescents were recruited in local public high schools in the US and the Netherlands. In the Netherlands, three schools participated in the area of Amsterdam and Haarlem (i.e., VMBO; preparatory middle-level applied education/MBO; middle-level applied education, vocational training). In the US, four schools in the New Haven County, Connecticut participated in the study. Information sheets detailing the study were mailed to all parents of students in the participating schools and parents were asked to inform the research team if they did not want their child to participate. Prior to any study procedures, assent was obtained from participants younger than 18 and consent from those who were 18 years old. All participants were provided with verbal instructions about all aspects of the study procedure and a detailed written instruction explaining the task prior to each computer task. The research assistant was present to answer questions if necessary; however they did not interrupt adolescents during the tasks. The order of the computer tasks was randomized. Subsequent to the computer tasks, participants completed questionnaires on demographic information, smoking behavior, and nicotine dependence. Participants received €5 (NL) or $25 (US) for participation. The study protocol was approved by the Ethical Committee of the Faculty of Social Sciences, University of Amsterdam, and the Institutional Review Board at Yale University School of Medicine and the participating schools.

### Measures

#### Approach–avoidance bias

The smoking approach and avoidance action tendencies were measured with an adapted version of the approach–avoidance task [AAT; (12, 24, 48)]. On this version of the smoking approach–avoidance task (S-AAT), participants viewed 20 tobacco-related images and 20 neutral images that were rotated 3° left or right. Each image was shown four times, twice rotated to the left and twice rotated to the right (i.e., 160 trials). Tobacco-related images were cigarettes, cigarette packages, and individuals smoking cigarettes. Neutral images were objects (e.g., a pen or pencil) or individuals matched to the tobacco-related images in terms of color and composition. Participants were instructed to push or pull the joystick in response to the rotation of the picture (i.e., left or right) and not the content (i.e., smoking or control). The smoking approach bias represented the relative difference between pushing and pulling the joystick in response to the smoking stimuli: being faster in pushing compared to pulling smoking stimuli indicated an avoidance bias and being faster in pulling compared to pushing indicated an approach bias. The task had a zooming feature: pushing and pulling the joystick gradually decreased and increased the image-size. Combined with arm flexion and extension movements, this feature mimicked approach and avoidance actions (12, 24, 48). Reaction time was logged when a complete pull or push response was made. A red cross would appear after a response error was made. To avoid order effects, half of the participants were instructed to pull images rotated left and push images rotated right and the other half vice versa. Prior to assessment, 15 practice trials were conducted (i.e., gray squares rotated left and right).

The pictures used in the S-AAT were validated by 6 adolescent smokers and 12 non-smokers in the US who rated 20 pairs of pictures on whether they looked realistic using a five-point Likert scale (1 = very unrealistic to 5 = very realistic). On average, all adolescents (regardless of smoking status) rated the pictures as looking at least somewhat realistic (*M* = 3.56, SD = 1.22). Smokers were also asked to rate each picture on how much it made them think about smoking (1 = not at all, 2 = somewhat, 3 = very much). All smoking images reminded smokers of cigarettes (77% reported “somewhat” and 25% reported “very much”) and non-smoking images generally did not make them think about cigarettes (77% reported “not at all” and 19% “somewhat” to “very much” that the non-smoking images made them think about cigarettes). The same pictures were used for the US and the Dutch studies. In the Netherlands, Joystick Pro Flight 2 (Logic 3) was used and the US used Logitech Attack 3.

#### Implicit memory associations

Implicit memory associations were assessed with a variety of the implicit association task [IAT; (49)]. The IAT is a computerized sorting task that infers implicit associations from the simultaneous classification of target categories and two affective attribute categories in two different sorting conditions. In the current study, a brief IAT version was used [i.e., bIAT; (50)]. It consisted of 13 blocks, in which participants were instructed to classify words as belonging or not belonging to a certain category [i.e., smoking, non-smoking, positive (e.g., excited, outgoing), negative (e.g., depressed, miserable), relaxed (e.g., rested, comfortable), and neutral (e.g., central, plain)] by pressing one of two keys on the keyboard (i.e., “e” = belong to category; “i” = does not belong to category). The bIAT consists of a bipolar (i.e., “positive” versus “negative” associations with smoking) and an unipolar (i.e., “relaxed” versus “neutral” associations with smoking) assessment. A total of eight versions were counterbalanced (i.e., eight different combinations of presented order of “smoking” – “positive” – “negative” – “relaxed” – “neutral”). For each category, three words were used. In the bipolar version, the target word (i.e., smoking, non-smoking) was combined with a bipolar sorting of the “positive” (e.g., excited, outgoing) and “negative” attributes (e.g., depressed, miserable). Hence, in one combined sorting condition “smoking” and “positive” shared a response-key, as did “smoking” and “negative.” In the unipolar version, the target word (i.e., smoking, non-smoking) was combined with “relaxed” (i.e., rested, comfortable) and “neutral” (i.e., central, plain) attributes. In one sorting condition, smoking-related targets shared a response with relaxed attributes, and in another with neutral attributes.

#### Attentional bias and inhibition skills

Attentional bias and inhibition skills were assessed in one combined task. Attentional bias was assessed with the emotional Stroop task (51–53). The task consisted of four blocks: (1) practice block (only symbols, colored letters below, 20 trials), (2) practice block (only symbols, without colored letters, 20 trials), (3) the emotional block (smoking-related and control words, 64 trials), and (4) the classic block (words and symbols, 28 trials). Within each block, the stimuli were randomized. The task measures whether individuals react slower to smoking-related words than to neutral words. First, participants practiced matching E, F, J, and I keyboard letters to the colors red, yellow, blue, and green on neutral signs (e.g., ####) during 40 trials. During the first 20 trials, the letter–color matching were visible at the bottom of the screen, while in the latter 20 trials the letter–color matching were invisible. Next, the assessment of attentional bias with the emotional block started. Here, eight smoking-related (e.g., tobacco, cigarette, smoke, and ashtray) and eight neutral words (e.g., arrival, clock, trophy, and nettle) were presented in each of the four colors resulting in 64 trials. Participants were instructed to indicate the color of the word as fast as possible. Next, the classical Stroop block was presented [cf. (54–56)], which consisted of 28 trials. The words used were: red, yellow, green, and blue. A word remained on the screen until a response or for a maximum of 4000 ms. During the inter-trial interval in both Stroop tasks, a fixation cross was shown for 1000 ms (i.e., there was no real “empty” inter-trial interval, just a repeated fixation period of 1000 ms).

#### Working memory

Working memory was assessed with self-ordering pointing task [SOPT; (57)]. The task consisted of eight different blocks with increasing difficulty where concrete pictures (e.g., flower, house, and elephant) were shown in a grid of 6, 8, 10, or 12 pictures. During each block, participants were shown a specific grid of pictures. Location of the pictures within this grid changed between trials. Participants were instructed to click a different picture on each trial by clicking on it with the mouse cursor until all pictures were clicked on without clicking on the same location twice in a row. The participants thereby needed to memorize, which picture they clicked on and where they had clicked the previous trial. No counterbalancing across participants was deemed necessary. The stimulus duration was 10,000 ms or until response. An inter-stimulus interval of 500 ms was used, after which the shuffled pictures were presented again.

#### Risk taking

Risk taking was assessed with the balloon analog risk task [BART; (58)]. Participants were shown a picture of a balloon, and instructed to inflate this balloon for an increasing reward (total of 20 trials). Inflating the balloon (0–128 pumps) increased the size of the balloon and the associated reward. However, an overinflated balloon would result in a blowout and the participant losing the reward of that trial. Each balloon had a different bursting point, on a scale of 1–128. We used a version of the BART that allowed participants to choose the intended number of pumps for that specific trial (59).

#### Smoking

Adolescent smokers answered an open-ended question on how many cigarettes they smoke a day. They also completed the modified Fagerström Tolerance Questionnaire, which assessed nicotine dependence [mFTQ; (60)]. The Cronbach’s α of this questionnaire was 0.59.

### Data preparation

#### Approach–avoidance bias (S-AAT)

To correct for outliers, reaction times (RTs) below 200 ms, above 2000 ms, and more than 3 SD above and below the individual mean were removed for each participant. Participants with excessive errors [>35%, Ref. (61)] were removed from the analytical sample. To calculate the bias score, mean approach RT was subtracted from mean avoid RT for each image category. The subtraction resulted in a bias score for tobacco and neutral images for each participant. We used mean scores for all analyses. A relatively faster approach compared to avoid RTs was indicated with a positive score, whereas a relatively faster avoid compared to approach RTs was indicated with a negative score.

#### Implicit associations (bIAT)

Scores were calculated with the D600 algorithm (62), so that more positive scores indicated relatively strong associations between “smoking” and “positive” and “smoking” and “relaxed.”

#### Attentional bias and inhibition skills (emotional and classical Stroop)

Trials with RTs below 200 ms and above 2000 ms were excluded from further analysis. Mean RTs were calculated for smoking and control categories. The Stroop interference score was calculated by subtracting the neutral RTs from the incongruent RTs with higher scores indicating weaker inhibition (i.e., inhibition skills).

#### Working memory (SOPT)

The total amount of errors of all trials was calculated as a measure of working memory. A proportion measure was calculated so that errors weigh more in the first blocks (in which there are less pictures).

#### Balloon analog risk task

The average number of pumps on all trials of 20 balloons was calculated. Unlike in the standard BART, in the automatic version participants state at the beginning of each trial how many pumps (risks) they wish to do and then observe the sequence of events unfold. By doing so biased risk scores are avoided as when for instance the desired number of balloon pumps exceeds the explosion point. Indeed in the standard BART, this bias is corrected in the adjusted average pumps score measure. However, when using the automatic BART there is no reason to use the adjusted average pumps (59).

Twenty-seven participants were removed due to excessive S-AAT errors and extreme scores on the IAT. Five participants reported smoking less than five cigarettes a day. These participants were also removed from the analytical sample. The analytical sample consisted of 56 Dutch adolescents (27 smokers and 29 non-smokers) and 37 American adolescents (19 smokers and 18 non-smokers). A chi square test of independence demonstrated that the number of exclusions based on errors did not differ significantly between the American and Dutch samples χ^2^(1, *N* = 125) = 3.08, *p* = 0.08.

## Results

### Descriptives

Participants (*n* = 46 smokers) smoked on average 11.89 (SD = 5.58) cigarettes a day and had a nicotine dependence score of 2.90 (SD = 1.36) indicating low-to-moderate dependence. Amount of daily cigarettes did not differ between the American and Dutch samples, however, American adolescents (*M* = 3.50; SD = 1.18) scored higher on nicotine dependence than did the Dutch (*M* = 2.47; SD = 1.35) *t*(41.62) = 2.73; *p* = 0.009. No significant differences were observed between smokers and non-smokers or American and Dutch samples on age and gender. In Table 1, correlations among model variables are displayed for smokers. Daily smoking was positively marginally correlated with nicotine dependence (*p* = 0.09) and risk taking (*p* = 0.04) and correlated negatively with working memory (*p* = 0.03). Nicotine dependence correlated positively with attentional bias (*p* = 0.02) and marginally with inhibition skills (*p* = 0.09) indicating that higher levels of nicotine dependence might be associated with stronger attentional bias and weaker inhibition skills. Finally, approach bias and positive associations with smoking were negatively correlated (*p* = 0.04).

**Table 1:** **Correlations between model variables**.

	1	2	3	4	5	6	7	8	9
1. Smoking	–								
2. mFTQ	0.27^+^	–							
3. Approach bias	0.04	0.05	–						
4. Positive ass.	0.11	0.02	−0.30*	–					
5. Relaxed ass.	0.03	−0.02	−0.03	0.21	–				
6. Attentional bias	0.01	0.34*	−0.04	0.06	−0.05	–			
7. Working memory	−0.35*	0.01	0.03	−0.03	−0.01	−0.09	–		
8. Inhibition skills	−0.01	0.26^#^	−0.01	−0.15	−0.14	−0.11	−0.05	–	
9. Risk taking	0.30*	−0.07	−0.10	−0.00	−0.07	0.00	−0.19	−0.06	–

*p < 0.01, **p < 0.001, ^+^*p* = 0.09, ^#^*p* = 0.09. Smoking, Amount of daily cigarettes; mFTQ, nicotine dependence. Approach bias, toward smoking stimuli (S-AAT), Positive ass., positive versus negative associations with smoking (bIAT), Relaxed ass., relaxed versus neutral associations with smoking (bIAT). Attentional bias, emotional Stroop. Working memory, SOPT. Inhibition skills, classical Stroop. Risk taking, BART. Correlations are among smokers only.

### Differences in approach–avoidance bias (S-AAT) between smokers and non-smokers

With a 2 × 2 × 2 mixed-design analysis of variance (ANOVA), differences between American and Dutch smokers and non-smokers in the approach–avoidance bias scores were examined. The within-factor was bias scores (i.e., neutral/smoking) and the between-factors were nationality (i.e., American adolescents/Dutch adolescents) and smoking status (i.e., smoker/non-smoker). There was a main-effect of bias scores (*p* = 0.02; ηρ^2^ = 0.06; neutral: *M* = −29.54, SE = 9.94, smoking: *M* = −10.34, SE = 9.15), indicating that participants had a stronger avoidance bias toward neutral pictures than toward smoking pictures. Also, there was an effect of nationality (*p* < 0.001; ηρ^2^ = 0.13) indicating that Dutch adolescents had an avoidance bias toward both smoking and neutral pictures (*M* = −51.23; SE = 10.87) and that American adolescents did not have a particular bias toward both smoking and neutral pictures (*M* = 11.34; SE = 13.37). One-sample *t*-tests for Dutch and American samples separately demonstrated that American adolescents generally had no bias toward smoking (*p* = 0.32) and neutral pictures (*p* = 0.62), whereas Dutch adolescents generally had an avoidance bias toward both smoking (*M* = −36.41, SD = 79.89; *p* < 0.001) and neutral (*M* = −65.96, SD = 99.63; *p* < 0.001) pictures. Further, there was no difference in biases between smokers and non-smokers (*p* = 0.90).

### Differences in attentional bias (emotional Stroop) between smokers and non-smokers

Color-naming RTs for smoking and control words were analyzed using a 2 × 2 × 2 mixed-design ANOVA. The within-factor was word type (i.e., smoking words/neutral words) and the between-factors were nationality (i.e., American/Dutch) and smoking status (i.e., smoker/non-smoker). Results demonstrated no significant effects of word type (*p* = 0.40), smoking status (*p* = 0.48), nationality (*p* = 0.86) and interaction between smoking status and word type (*p* = 0.87), and nationality and word type (*p* = 0.74).

### Differences in implicit associations (bIAT) between smokers and non-smokers

Differences in implicit associations between smokers and non-smokers were analyzed with an ANOVA separately for “positive” versus “negative” and “relaxed” versus “neutral” associations. For “positive” versus “negative” associations, no significant main-effect of smoking status (*p* = 0.59) or nationality (*p* = 0.35) was found. The interaction between nationality and smoking status was also not significant (*p* = 0.74). For the “relaxed” versus “neutral” bias, there was also no main-effect of smoking status (*p* = 0.63) or nationality (*p* = 0.44). The interaction between smoking status and nationality was also not significant (*p* = 0.73).

### Do cognitive biases (S-AAT, emotional Stroop, bIAT), risk taking (BART), inhibition skills (classical Stroop), and working memory (SOPT) predict smoking behavior?

To detect whether the cognitive biases [i.e., approach bias (S-AAT), attentional bias (emotional Stroop) and implicit associations (bIAT)], risk taking (BART), inhibition skills (classical Stroop), and working memory (SOPT) predicted nicotine dependence and daily smoking within the group of smokers, two separate hierarchical regressions were conducted (i.e., one for nicotine dependence and one for daily smoking). In the first step, the neutral bias score (i.e., S-AAT) and neutral word score on the emotional Stroop were added to control for general biases. Nationality was also entered as a covariate in the analysis regarding nicotine dependence. In the second step, the approach bias toward smoking (i.e., S-AAT), emotional Stroop smoking word score, “positive” versus “negative” associations, “relaxed” versus “neutral” associations, risk taking, inhibition skills, and working memory were added.

The results showed that within the group of smokers, attentional bias, and inhibition skills predicted nicotine dependence [*F*(10, 41) = 3.21, *p* < 0.01], demonstrating that stronger attentional bias and weaker inhibition skills were related to higher level of nicotine dependence (Table 2; maximum Cook’s distance = 0.14, maximum standardized residual = 2.18, multicollinearity Tolerance above = 0.10). Although the ANOVA *F*-test was significant for the second step in the hierarchical regression, the *R*^2^ change was not significant.

**Table 2:** **Hierarchical multiple regression analyses predicting daily cigarette use and nicotine dependence within the group of smokers**.

	Smoking			Dependence		
	*B*	SE *B*	β	*B*	SE *B*	β
**STEP 1**						
Nationality				1.28	0.41	0.46**
Neutral bias (S-AAT)	0.01	0.01	0.15	− 0.00	0.00	− 0.22
Neutral bias (Stroop)	0.00	0.01	0.08	0.00	0.00	0.34*
**STEP 2**						
Approach bias (S-AAT)	0.01	0.02	0.16	0.00	0.00	0.11
Attentional bias (e. Stroop)	−0.00	0.01	− 0.08	0.01	0.00	0.67*
Positive associations (IAT)	2.63	2.20	0.22	0.08	0.44	0.03
Relaxed associations (IAT)	−0.95	1.98	− 0.08	− 0.15	0.39	− 0.05
Working memory (SOPT)	−21.00	11.62	− 0.30^+^	− 0.20	2.32	− 0.01
Inhibition skills (c. Stroop)	0.00	0.01	0.02	0.00	0.00	0.32*
Risk taking (BART)	0.11	0.07	0.28	0.00	0.01	0.01
*R*^2^ (step 1)		0.03			0.32**	
*ΔR*^2^ (step 2)		0.22			0.20	

*p < 0.05, **p < 0.01, ^+^*p* = 0.08. Neutral bias S-AAT, non-smoking control photos; neutral bias Stroop, non-smoking words.

Regarding the results of daily smoking, the model was not significant [*F*(6, 274.99) = 1.41, *p* = 0.22]. A trimmed model only including risk taking and working memory (since these two factors almost reached significance in the original model) was significant [*F*(2, 249) = 4.40, *p* < 0.05] with an explained variance of 0.19 (*p* < 0.05). Higher levels of risk taking was marginally associated with higher levels of daily smoking (β = 0.27, *p* = 0.08) and lower working memory was marginally associated with higher levels of daily smoking (β = −0.29, *p* = 0.06) (Table 2; maximum Cook’s distance = 0.16, maximum standardized residual = 2.94, multicollinearity Tolerance above = 0.10).

## Discussion

The present study is the first to examine cognitive smoking biases in adolescent smokers and non-smokers. Contrary to our expectations, attentional bias, approach bias, and implicit associations toward smoking-related stimuli did not differ between smokers and non-smokers. Unexpectedly, there was a difference in approach bias toward both smoking and non-smoking stimuli between the American and Dutch adolescents independent of smoking status. The Dutch adolescents generally had an avoidance bias to both neutral and smoking stimuli, whereas the American adolescents generally did not have a bias. Within the group of smokers, American adolescents demonstrated higher levels of nicotine dependence than Dutch adolescents. Moreover, even after controlling for nationality, adolescent smokers with stronger attentional bias and weaker inhibition skills showed higher levels of nicotine dependence. Therefore, attentional bias appears to be an important factor in nicotine dependence, whereas positive and relaxed associations with smoking and approach bias do not appear to be underlying motivational constructs driving smoking behavior in this sample of adolescents. This is not in line with findings in young adults and adults where these biases have been found regarding smoking (20, 22, 29, 32, 33). Our data also suggest that characteristics related to executive functioning such as weak inhibition skills, higher levels of risk taking, and low levels of working memory might be more important in the prediction of smoking behavior in adolescence. That is because in a trimmed model, risk taking and working memory were related to amount of daily cigarettes smoked.

Although attentional bias, approach bias, and implicit associations did not differ between smokers and non-smokers, attentional bias was related to nicotine dependence within the group of smokers. This is in line with a study on reward-related attentional processes and alcohol use in adolescence (38) and among smokers between 18 and 40 years (18). Our study indicates that in adolescents, stronger attentional bias might be associated with higher level of nicotine dependence corresponding with the incentive–sensitization theory according to which repeated drug use heightens the incentive salience of cues associated with using that particular drug, which in turn increase attention toward such cues (11). However, this result does not match those of Mogg and colleagues (22), who found that young adult smokers with *lower* level of nicotine dependence had stronger attentional bias and faster approach responses to smoking-related stimuli, in line with earlier findings (63, 64) indicating that lighter smokers demonstrated greater attentional bias for smoking cues than heavier smokers. These findings were interpreted from an “incentive-habit” model (65), which states that incentive salience is important in the first escalation phase of addiction, and later habit processes become more important [cf. (66)]. However, as the current sample was low-to-moderate nicotine dependent, our findings might support the case that lighter smokers demonstrate greater attentional bias. Future studies should investigate cognitive biases in heavy and light adolescent smokers to be able to disentangle this.

Moreover, there are several other factors related to smoking that could affect the relationship between cognitive biases and smoking behavior. For instance, other indicators of dependence such as previous attempts to quit smoking might influence the relationship between cognitive biases and smoking behavior [Ref. (31, 63), no bias in successful ex-smokers]. However, adding number of quit attempts as a covariate in the current models within the group of smokers showed comparable results. Another factor may be craving. A recent study demonstrated that the level of craving is related to the approach bias and that smoking a cigarette led to reduced craving but an increased approach bias (30). Although this finding is difficult to interpret, future studies should examine this relationship more thoroughly. Finally, in a study where craving was increased by having participants abstinent 2 h prior to participation, heavy smokers had an approach bias whereas abstinent heavy smokers did not (31). Unfortunately, we did not assess craving in the current study. These studies were all conducted on young adults and adults; future studies should examine how attempts to quit, craving, and heavy smoking relate to cognitive biases in adolescents.

In the current study, we found that within the group of smokers, attentional bias might be more important than the approach bias and implicit memory associations. Other factors may contribute to the lack of findings of cognitive biases in adolescent smokers compared to (young) adult smokers where the biases have been found (20, 22, 29, 32, 33). Legislation may also play a role in the findings of the current study. For (young) adults, it is legal to use cigarettes. However, the American adolescents are underage as in the US the minimum age of buying cigarettes is 18 years. In the Netherlands, from the first of January 2014, the minimum age of buying cigarettes has been raised from 16 to 18 years (http://www.rijksoverheid.nl/nieuws/2013/11/12/leeftijdsgrens-tabaksverkoop-naar-18-jaar-vanaf-1-januari-2014.html). During the years preceding that change, there have been campaigns aiming at a norm change toward smoking. These campaigns have focused on both adolescent smokers and non-smokers to make it less hip for youth to smoke. Also, Dutch studies have showed that smoke-free legislation increased smoking cessation [e.g., Ref. (67)]. Although speculative, these norm changes and changes in legislation might influence cognitions related to smoking in adolescents. Our finding of avoidance bias toward smoking-related stimuli in the Dutch sample could be explained by a general norm change toward smoking. This is an interesting finding because it indicates that the smoking-related pictures were not attractive, not even among smokers. However, this should be interpreted with caution as the Dutch participants were also negative toward the control stimuli.

Nevertheless, within the group of smokers, in addition to attentional bias, inhibition skills were associated with level of nicotine dependence and working memory and risk taking may be related to amount of cigarettes smoked daily. Although interpretation should be cautious, these behavioral impulsivity related constructs might thus be important factors related to smoking behavior in both American and Dutch adolescents, which is in line with previous studies [e.g., college students: (68); adolescents: (69–71)]. An important aspect of nicotine dependence is the inability to quit smoking despite the awareness of the negative consequences. The amount of cigarettes that someone smokes might not necessarily say something about the level of dependence. Our results suggest that the attentional bias is related to the severity of the dependence rather than the absolute amount that someone uses, which in turn support the importance of the attentional bias in substance use disorders.

Moreover, recent studies indicate that relatively poor inhibition skills moderate the relation between automatic processes related to drinking and actual drinking in at-risk adolescents cross-sectionally (26) and prospectively (27). In the current study, we did not have sufficient power to investigate such interactions. Nevertheless, this is important to examine in future studies as the associations between cognitive biases and smoking behavior may be stronger when working memory and inhibition skills are poor and risk taking is high [e.g., Ref. (28, 54, 72)].

Some strengths of the current study are the bi-national approach studying automatic smoking cognitions in adolescents. Moreover, the study also included behavioral assessments of risk taking, inhibition skills, and working memory, which allowed studying the relative predictive value of cognitive biases and constructs related to executive functioning and risk taking on smoking in adolescents. Despite these strengths, this study also has some limitations. The fact that Dutch adolescents had avoidance bias toward both the control and the smoking stimuli emphasizes the importance of validating the stimuli in both countries. In this study, only the American adolescents evaluated the stimuli, which were then used on both nations. However, we found it crucial to use the same stimuli in both countries and therefore chose to use the pictures selected by the American pilot group in the Dutch study as well. Given that attentional bias and implicit associations did not differ between the American and Dutch adolescents, the difference might be caused by the stimuli used due to lack of ecological validity for the Dutch adolescents. Secondly, there were no specific instructions as to whether or not participants could smoke prior to participating in the study. Factors such as withdrawal symptoms and cravings to smoke may have affected their responses. However, we did not assess them in this study so we could not take this into account in the analyses. Also, as studies have found that peers influence individual smoking behavior [e.g., Ref. (73, 74)], it may be important to combine implicit assessments with the adolescents’ social context. Thirdly, other substance use such as alcohol and marihuana might also be associated with executive functioning in adolescence. Future studies with larger samples should investigate these relationships. Fourthly, the reimbursements differed across sites. The reimbursements are based on time the participant spent on the research. This amount, however, differs between the two countries but within each country, the amounts were similar to what participants usually would receive in this age group. In this case, participants were paid for completing the tasks including the AAT during this time period. They were not told that the payment was for completing the AAT or performing optimally on the AAT. If that had been the case of course we would be worried that the payment would alter their motivation to participate. Finally, our findings are preliminary and we recommend future studies to replicate these findings with a larger sample so that subgroups of smokers (e.g., high versus low nicotine dependence or low versus high craving levels) and interactions with for instance executive functioning can be examined.

## Conclusion

In sum, the present study did not find differences in cognitive biases between adolescent American and Dutch smokers and non-smokers. Although, these results are tentative due to small sample size, within the group of smokers, it appears that attentional bias and inhibition skills may be important factors for adolescent nicotine dependence and lower levels of working memory as well as higher risk taking might be important factors in explaining daily smoking. Future studies should investigate possible cultural differences in approach–avoidance bias using larger samples.

## Author Contributions

Helle Larsen, Grace Kong, Daniela Becker, Dana Cavallo, Suchitra Krishnan-Sarin, and Reinout Wiers contributed to the design of the experiments and procedure on the US and NL site, respectively. Helle Larsen and Grace Kong contributed equally to the study and this manuscript and are therefore listed as shared first author. Suchitra Krishnan-Sarin and Reinout Wiers also contributed equally and are listed as shared last author. Janna Cousijn developed the S-AAT task and assisted with scoring of S-AAT data and analyses. Wouter Boendermaker developed the BART, emotional and classical Stroop task and SOPT task. Helle Larsen and Daniela Becker managed and collected data at the Dutch site and Grace Kong and Dana Cavallo managed and collected data at the US site. Helle Larsen and Daniela Becker scored the data on the behavioral assessments. Helle Larsen and Grace Kong conducted all analyses and drafted the manuscript. All authors contributed in revising and improving the manuscript. Helle Larsen did the final approval of the version to be published. All authors agree to be accountable for these aspects of the work.

## Conflict of Interest Statement

The authors declare that the research was conducted in the absence of any commercial or financial relationships that could be construed as a potential conflict of interest.
